# Supplementary material for: Quantitative Proteomics and Phosphoproteomics Support a Role for Mut9-Like Kinases in Multiple Metabolic and Signaling Pathways in Arabidopsis
Source: Mol Cell Proteomics. 2021 Mar 5;20:100063. doi: 10.1016/j.mcpro.2021.100063 (PMC8066427; doi:10.1016/j.mcpro.2021.100063)
Supplement: Supplemental Figures S1–S2 and Tables S1–S3 [file mmc1.pdf]

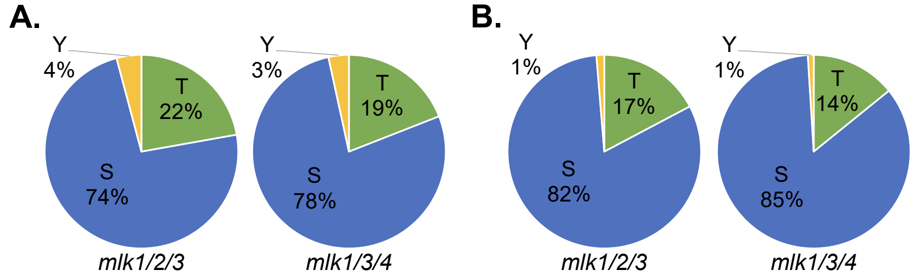

Supplemental Figure S1. Phosphosite distribution of differentially phosphorylated peptides in *mlk* mutants

The of threonine (T) phosphorylation, serine (S) phosphorylation, and tyrosine (Y) phosphorylation sites identified as having altered abundance in *mlk1/2/3* or *mlk1/3/4* mutants compared to wildtype samples at ZT12 (A) or ZT14 (B).

A.

	#	Motif	Motif Score	Foreground Matches	Foreground Size	Background Matches	Background Size	Fold Increase
Decreased	1	....R..S.......	5.08	12	48	55686	1013104	4.55
Increased	1	.......SP......	5.71	11	37	53111	1013104	5.67

B.

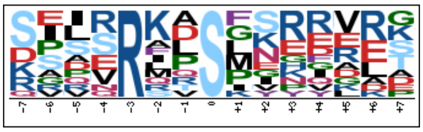

C.

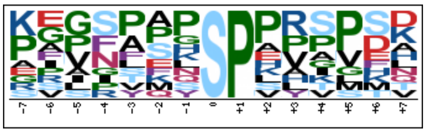

Supplemental Figure S2. Motif-X analysis of phosphopeptides with decreased abundance in *mlk1/2/3* mutants.

Phosphopeptides with altered abundance in *mlk1/2/3* (ZT12) mutants where extended and aligned using PEPTIDEXTENDER (http://schwartzlab.uconn.edu/pepextend). Motif-X analysis was then preformed with the probability threshold was set to p-value ≤ 10^{-6}, the occurrence threshold was set to 10. The default IPI Arabidopsis Proteome data set was used as the background data set.

Supplemental Table S1. Summary of Pairwise Comparisons

	Global Proteome			Phosphoproteome		
Pairwise Comparison	Total Peptides with Altered Abundance	Proteins with Increase Abundance	Proteins with Decrease Abundance	Total Peptides with Altered Abundance	Proteins with Increase Abundance	Proteins with Decrease Abundance
*mlk123*_ZT12/ WT_ZT12	15	0	13	113	44	50
*mlk134*_ZT12/ WT_ZT12	232	28	95	429	103	190
*mlk123*_ZT14/ WT_ZT14	20	6	7	170	87	63
*mlk134*_ZT14/ WT_ZT14	228	76	36	306	108	115

Supplemental Table S2. REVIGO Cellular Component GO-term Enrichment Analysis

***mlk1/2/3* ZT12**

GO-term ID	Description	Frequency	Log_{10} p-value	Uniqueness	Dispensability
**GO:0005654**	**nucleoplasm**	**1.37%**	**-3.0182**	**0.15**	**0**
GO:0044451	*nucleoplasm part*	*0.90%*	*-2.0482*	*0.17*	*0.82*
**GO:0043226**	**organelle**	**20.79%**	**-2.5969**	**0.75**	**0**
**GO:0005634**	**nucleus**	**8.97%**	**-4.4283**	**0.13**	**0.51**
GO:0043231	*intracellular membrane-bounded organelle*	*13.76%*	*-2.6364*	*0.12*	*0.75*
**GO:0043227**	**membrane-bounded organelle**	**14.45%**	**-3.382**	**0.17**	**0.57**
GO:0043229	*intracellular organelle*	*19.92%*	*-2.6144*	*0.16*	*0.7*

***mlk1/3/4* ZT12**

GO-term ID	Description	Frequency	Log_{10} p-value	Uniqueness	Dispensability
**GO:0005575**	**cellular_component**	**100.00%**	**-1.7986**	**1**	**0**
**GO:0005623**	**cell**	**53.55%**	**-12.1624**	**0.97**	**0**
**GO:0005654**	**nucleoplasm**	**1.37%**	**-13.8069**	**0.31**	**0**
GO:0044451	*nucleoplasm part*	*0.90%*	*-10.1113*	*0.33*	*0.82*
GO:0070013	*intracellular organelle lumen*	*2.74%*	*-8.9747*	*0.3*	*0.95*
GO:0031981	*nuclear lumen*	*2.29%*	*-10.58*	*0.29*	*0.87*
GO:0043233	*organelle lumen*	*2.74%*	*-8.9747*	*0.33*	*0.97*
**GO:0031974**	**membrane-enclosed lumen**	**2.74%**	**-8.9747**	**0.94**	**0**
**GO:0032991**	**macromolecular complex**	**14.01%**	**-3.6517**	**0.95**	**0**
**GO:0043226**	**organelle**	**20.79%**	**-11.9788**	**0.95**	**0**
**GO:0043234**	**protein complex**	**6.42%**	**-4.0467**	**0.88**	**0**
**GO:0005829**	**cytosol**	**2.55%**	**-6.6478**	**0.72**	**0.17**
**GO:0044424**	**intracellular part**	**35.65%**	**-11.7077**	**0.68**	**0.22**
**GO:0005622**	**intracellular**	**41.18%**	**-11.4711**	**0.77**	**0.32**
**GO:0044444**	**cytoplasmic part**	**12.66%**	**-2.011**	**0.66**	**0.35**
**GO:0044464**	**cell part**	**52.39%**	**-10.7959**	**0.77**	**0.38**
**GO:0043227**	**membrane-bounded organelle**	**14.45%**	**-12.9172**	**0.47**	**0.4**

GO:0043229	*intracellular organelle*	*19.92%*	*-11.5935*	*0.39*	*0.7*
**GO:0000785**	**chromatin**	**0.47%**	**-9.0726**	**0.41**	**0.46**
GO:0044427	*chromosomal part*	*1.12%*	*-7.4962*	*0.39*	*0.84*
GO:0000790	*nuclear chromatin*	*0.23%*	*-1.6216*	*0.34*	*0.78*
**GO:0005694**	**chromosome**	**1.51%**	**-6.6556**	**0.5**	**0.49**
**GO:0005652**	**nuclear lamina**	**0.00%**	**-2.2358**	**0.53**	**0.51**
**GO:0043228**	**non-membrane-bounded organelle**	**8.41%**	**-2.4597**	**0.5**	**0.56**
**GO:0044428**	**nuclear part**	**3.12%**	**-10.7447**	**0.37**	**0.57**
GO:0044446	*intracellular organelle part*	*8.94%*	*-7.9355*	*0.32*	*0.73*
**GO:0044422**	**organelle part**	**9.43%**	**-7.8861**	**0.5**	**0.58**
**GO:0005634**	**nucleus**	**8.97%**	**-19.4547**	**0.39**	**0.58**
GO:0043231	*intracellular membrane-bounded organelle*	*13.76%*	*-12.6216*	*0.37*	*0.75*
**GO:0031965**	**nuclear membrane**	**0.10%**	**-1.7721**	**0.46**	**0.6**
**GO:0005849**	**mRNA cleavage factor complex**	**0.05%**	**-1.71**	**0.42**	**0.62**
**GO:0016607**	**nuclear speck**	**0.09%**	**-3.6498**	**0.42**	**0.65**
GO:0016604	*nuclear body*	*0.19%*	*-2.4895*	*0.39*	*0.7*
**GO:0043232**	**intracellular non-membrane-bounded organelle**	**7.95%**	**-2.4597**	**0.41**	**0.67**

mlk1/2/3 **ZT14**

GO-term ID	Description	Frequency	Log_{10} p-value	Uniqueness	Dispensability
**GO:0005623**	**cell**	**53.55%**	**-1.3298**	**0.93**	**0**
**GO:0031974**	**membrane-enclosed lumen**	**2.74%**	**-1.5258**	**0.87**	**0**
**GO:0044427**	**chromosomal part**	**1.12%**	**-3.9547**	**0.26**	**0**
GO:0000785	*chromatin*	*0.47%*	*-1.9872*	*0.27*	*0.84*
GO:1902494	catalytic complex	3.73%	-3.6925	0.82	0
**GO:0044422**	**organelle part**	**9.43%**	**-1.5686**	**0.35**	**0.36**
**GO:0043228**	**non-membrane-bounded organelle**	**8.41%**	**-1.3565**	**0.36**	**0.51**
**GO:0031981**	**nuclear lumen**	**2.29%**	**-2.757**	**0.23**	**0.53**
GO:0043233	*organelle lumen*	*2.74%*	*-1.5258*	*0.24*	*0.97*
GO:0070013	*intracellular organelle lumen*	*2.74%*	*-1.5258*	*0.23*	*0.95*

**GO:0005694**	**chromosome**	**1.51%**	**-2.9914**	**0.37**	**0.54**
**GO:0005634**	**nucleus**	**8.97%**	**-2.8239**	**0.32**	**0.55**
**GO:0044428**	**nuclear part**	**3.12%**	**-2.063**	**0.27**	**0.61**
GO:0044446	*intracellular organelle part*	*8.94%*	*-1.5918*	*0.22*	*0.73*
**GO:0043232**	**intracellular non-membrane-bounded organelle**	**7.95%**	**-1.3565**	**0.28**	**0.67**

***mlk1/3/4* ZT14**

GO-term ID	Description	Frequency	Log_{10} p-value	Uniqueness	Dispensability
**GO:0005623**	**cell**	**53.55%**	**-5.2218**	**0.95**	**0**
**GO:0005654**	**nucleoplasm**	**1.37%**	**-6.6778**	**0.29**	**0**
GO:0043233	*organelle lumen*	*2.74%*	*-2.4559*	*0.31*	*0.97*
GO:0044451	*nucleoplasm part*	*0.90%*	*-4.9706*	*0.31*	*0.82*
GO:0070013	*intracellular organelle lumen*	*2.74%*	*-2.4559*	*0.27*	*0.95*
GO:0031981	*nuclear lumen*	*2.29%*	*-2.9547*	*0.26*	*0.87*
**GO:0031974**	**membrane-enclosed lumen**	**2.74%**	**-2.4559**	**0.91**	**0**
**GO:0043226**	**organelle**	**20.79%**	**-4.0615**	**0.92**	**0**
**GO:0044464**	**cell part**	**52.39%**	**-4.4935**	**0.72**	**0.12**
**GO:0005829**	**cytosol**	**2.55%**	**-3.8268**	**0.67**	**0.17**
**GO:0044424**	**intracellular part**	**35.65%**	**-3.8386**	**0.62**	**0.35**
**GO:0005622**	**intracellular**	**41.18%**	**-3.52**	**0.71**	**0.38**
**GO:0044427**	**chromosomal part**	**1.12%**	**-3.1024**	**0.36**	**0.5**
GO:0000785	*chromatin*	*0.47%*	*-1.8861*	*0.4*	*0.84*
**GO:0070449**	**elongin complex**	**0.01%**	**-1.719**	**0.48**	**0.53**
**GO:0005694**	**chromosome**	**1.51%**	**-2.1232**	**0.47**	**0.54**
**GO:0044428**	**nuclear part**	**3.12%**	**-1.857**	**0.33**	**0.57**
**GO:0005634**	**nucleus**	**8.97%**	**-8.9136**	**0.35**	**0.58**
GO:0043231	*intracellular membrane-bounded organelle*	*13.76%*	*-3.3686*	*0.33*	*0.75*
**GO:0043229**	**intracellular organelle**	**19.92%**	**-4.091**	**0.35**	**0.62**
GO:0043227	*membrane-bounded organelle*	*14.45%*	*-3.6861*	*0.44*	*0.7*

Supplemental Table S3. REVIGO Biological Process GO-term Enrichment Analysis

***mlk1/2/3* ZT12**

GO-term ID	Description	Frequency	Log_{10} p-value	Uniqueness	Dispensability
**GO:0040029**	**regulation of gene expression, epigenetic**	**0.13%**	**-1.4572**	**1**	**0**

***mlk1/3/4* ZT12**

GO-term ID	Description	Frequency	Log_{10} p-value	Uniqueness	Dispensability
**GO:0006325**	**chromatin organization**	**0.67%**	**-10.4078**	**0.88**	**0**
GO:0016043	*cellular component organization*	*7.24%*	*-2.5575*	*0.86*	*0.75*
GO:0007064	*mitotic sister chromatid cohesion*	*0.05%*	*-2.1267*	*0.89*	*0.54*
GO:0051276	*chromosome organization*	*1.48%*	*-10.1433*	*0.87*	*0.53*
GO:0016570	*histone modification*	*0.37%*	*-2.0696*	*0.77*	*0.9*
GO:0016569	*covalent chromatin modification*	*0.42%*	*-4.2168*	*0.8*	*0.7*
GO:0006996	*organelle organization*	*3.60%*	*-4.5768*	*0.86*	*0.65*
**GO:0007623**	**circadian rhythm**	**0.06%**	**-1.6968**	**0.98**	**0**
**GO:0009987**	**cellular process**	**63.78%**	**-1.6536**	**0.99**	**0**
**GO:0010033**	**response to organic substance**	**0.90%**	**-4.1475**	**0.76**	**0**
GO:0001101	*response to acid chemical*	*0.12%*	*-1.5017*	*0.79*	*0.59*
GO:1901701	*cellular response to oxygen-containing compound*	*0.35%*	*-2.6778*	*0.71*	*0.8*
GO:1901700	*response to oxygen-containing compound*	*0.50%*	*-1.9101*	*0.77*	*0.68*
GO:0097305	*response to alcohol*	*0.06%*	*-3.15*	*0.76*	*0.55*
GO:0009737	*response to abscisic acid*	*0.03%*	*-3.224*	*0.77*	*0.67*
GO:0009725	*response to hormone*	*0.34%*	*-2.3585*	*0.74*	*0.79*
GO:0071359	*cellular response to dsRNA*	*0.02%*	*-1.8794*	*0.73*	*0.81*
GO:0033993	*response to lipid*	*0.23%*	*-2.6289*	*0.74*	*0.77*
GO:0070887	*cellular response to chemical stimulus*	*1.01%*	*-2.4437*	*0.72*	*0.73*
GO:0043331	*response to dsRNA*	*0.03%*	*-1.8794*	*0.76*	*0.83*
GO:0071407	*cellular response to organic cyclic compound*	*0.17%*	*-2.6615*	*0.7*	*0.67*
GO:0071310	*cellular response to organic substance*	*0.65%*	*-1.9393*	*0.69*	*0.87*

GO:0014070	*response to organic cyclic compound*	*0.23%*	*-2.821*	*0.74*	*0.75*
**GO:0010605**	**negative regulation of macromolecule metabolic process**	**1.17%**	**-5.5421**	**0.51**	**0**
GO:0031050	*dsRNA fragmentation*	*0.02%*	*-1.9355*	*0.59*	*0.98*
GO:0031047	*gene silencing by RNA*	*0.09%*	*-4.1379*	*0.56*	*0.75*
GO:0048523	*negative regulation of cellular process*	*1.83%*	*-2.0635*	*0.57*	*0.92*
GO:0031324	*negative regulation of cellular metabolic process*	*1.17%*	*-3.3862*	*0.52*	*0.97*
GO:0031327	*negative regulation of cellular biosynthetic process*	*0.77%*	*-4.1463*	*0.51*	*0.99*
GO:0035194	*posttranscriptional gene silencing by RNA*	*0.03%*	*-3.3893*	*0.59*	*0.84*
GO:0035196	*production of miRNAs involved in gene silencing by miRNA*	*0.01%*	*-2.5287*	*0.41*	*0.94*
GO:0035195	*gene silencing by miRNA*	*0.02%*	*-2.8097*	*0.56*	*0.97*
GO:0051253	*negative regulation of RNA metabolic process*	*0.63%*	*-1.5129*	*0.47*	*0.96*
GO:0070918	*production of small RNA involved in gene silencing by RNA*	*0.02%*	*-1.9355*	*0.41*	*0.98*
GO:2000113	*negative regulation of cellular macromolecule biosynthetic process*	*0.72%*	*-3.4461*	*0.49*	*0.98*
GO:0045934	*negative regulation of nucleobase-containing compound metabolic process*	*0.70%*	*-1.8539*	*0.49*	*0.92*
GO:0010629	*negative regulation of gene expression*	*0.78%*	*-5.1073*	*0.51*	*0.93*
GO:0009892	*negative regulation of metabolic process*	*1.26%*	*-4.8962*	*0.57*	*0.87*
GO:0009890	*negative regulation of biosynthetic process*	*0.77%*	*-4.0535*	*0.54*	*0.93*
GO:0010558	*negative regulation of macromolecule biosynthetic process*	*0.74%*	*-4.4935*	*0.5*	*0.92*
GO:0016458	*gene silencing*	*0.17%*	*-4.8125*	*0.55*	*0.8*
GO:0016441	*posttranscriptional gene silencing*	*0.03%*	*-2.7447*	*0.59*	*0.84*
GO:0051172	*negative regulation of nitrogen compound metabolic process*	*0.79%*	*-3.8477*	*0.53*	*0.93*
**GO:0032502**	**developmental process**	**2.81%**	**-2.6946**	**0.98**	**0**

**GO:0048511**	**rhythmic process**	**0.08%**	**-1.4271**	**0.98**	**0**
**GO:0048856**	**anatomical structure development**	**2.54%**	**-2.4547**	**0.97**	**0**
GO:0048731	*system development*	*1.26%*	*-1.342*	*0.95*	*0.86*
**GO:0050896**	**response to stimulus**	**12.21%**	**-4.4584**	**0.98**	**0**
**GO:0065007**	**biological regulation**	**20.50%**	**-8.3458**	**0.99**	**0**
**GO:0071840**	**cellular component organization or biogenesis**	**8.57%**	**-1.5986**	**0.98**	**0**
**GO:0046483**	**heterocycle metabolic process**	**29.66%**	**-5.9066**	**0.85**	**0.05**
**GO:0006807**	**nitrogen compound metabolic process**	**38.74%**	**-2.7423**	**0.94**	**0.06**
**GO:1901360**	**organic cyclic compound metabolic process**	**30.32%**	**-4.8928**	**0.89**	**0.08**
**GO:0006396**	**RNA processing**	**3.21%**	**-4.2725**	**0.68**	**0.14**
**GO:0018193**	**peptidyl-amino acid modification**	**1.50%**	**-2.2958**	**0.8**	**0.18**
**GO:0043170**	**macromolecule metabolic process**	**39.49%**	**-2.4622**	**0.88**	**0.21**
**GO:0010467**	**gene expression**	**19.67%**	**-5.3325**	**0.81**	**0.22**
**GO:0006725**	**cellular aromatic compound metabolic process**	**29.63%**	**-5.3325**	**0.85**	**0.24**
**GO:0097298**	**regulation of nucleus size**	**0.00%**	**-2.8327**	**0.82**	**0.28**
**GO:0048519**	**negative regulation of biological process**	**1.98%**	**-3.7352**	**0.74**	**0.32**
**GO:0048518**	**positive regulation of biological process**	**1.74%**	**-2.1864**	**0.74**	**0.33**
**GO:0040029**	**regulation of gene expression, epigenetic**	**0.13%**	**-4.4157**	**0.68**	**0.34**
**GO:0006397**	**mRNA processing**	**0.56%**	**-4.266**	**0.7**	**0.36**
GO:0008380	*RNA splicing*	*0.41%*	*-2.5214*	*0.71*	*0.62*
GO:0000377	*RNA splicing, via transesterification reactions with bulged adenosine as nucleophile*	*0.32%*	*-1.8729*	*0.71*	*0.99*
GO:0000375	*RNA splicing, via transesterification reactions*	*0.32%*	*-1.7721*	*0.71*	*0.95*
GO:0000398	*mRNA splicing, via spliceosome*	*0.32%*	*-2.266*	*0.7*	*0.88*
**GO:0009719**	**response to endogenous stimulus**	**0.53%**	**-2.1871**	**0.83**	**0.37**
**GO:0016071**	**mRNA metabolic process**	**0.80%**	**-3.0283**	**0.75**	**0.37**

**GO:0090304**	**nucleic acid metabolic process**	**21.45%**	**-5.9706**	**0.66**	**0.39**
GO:0032774	*RNA biosynthetic process*	*10.93%*	*-3.317*	*0.61*	*0.6*
GO:0018130	*heterocycle biosynthetic process*	*17.39%*	*-2.9957*	*0.71*	*0.52*
GO:0016070	*RNA metabolic process*	*15.95%*	*-5.0969*	*0.65*	*0.58*
GO:0006351	*transcription, DNA-templated*	*10.66%*	*-2.7986*	*0.58*	*0.74*
GO:0097659	*nucleic acid-templated transcription*	*10.72%*	*-3.0814*	*0.6*	*0.88*
GO:0006139	*nucleobase-containing compound metabolic process*	*26.55%*	*-5.9747*	*0.7*	*0.6*
GO:0019438	*aromatic compound biosynthetic process*	*16.95%*	*-2.7258*	*0.72*	*0.52*
GO:1901362	*organic cyclic compound biosynthetic process*	*17.87%*	*-2.5017*	*0.73*	*0.51*
GO:0034654	*nucleobase-containing compound biosynthetic process*	*14.53%*	*-2.7167*	*0.66*	*0.71*
**GO:0048583**	**regulation of response to stimulus**	**1.12%**	**-2.6925**	**0.63**	**0.4**
**GO:0044260**	**cellular macromolecule metabolic process**	**34.28%**	**-3.4685**	**0.74**	**0.43**
**GO:0034641**	**cellular nitrogen compound metabolic process**	**34.14%**	**-4.1543**	**0.77**	**0.44**
**GO:0050789**	**regulation of biological process**	**19.37%**	**-7.8794**	**0.66**	**0.45**
GO:0080090	*regulation of primary metabolic process*	*11.68%*	*-6.5952*	*0.55*	*0.87*
GO:0010468	*regulation of gene expression*	*10.82%*	*-5.2541*	*0.49*	*0.86*
GO:0031323	*regulation of cellular metabolic process*	*11.66%*	*-4.2907*	*0.52*	*0.87*
GO:0031326	*regulation of cellular biosynthetic process*	*10.82%*	*-4.4377*	*0.49*	*0.89*
GO:0051252	*regulation of RNA metabolic process*	*10.03%*	*-3.3936*	*0.42*	*0.86*
GO:2001141	*regulation of RNA biosynthetic process*	*9.97%*	*-2.9469*	*0.41*	*0.9*
GO:0019219	*regulation of nucleobase-containing compound metabolic process*	*10.26%*	*-3.7799*	*0.46*	*0.85*
GO:2000112	*regulation of cellular macromolecule biosynthetic process*	*10.68%*	*-4.1681*	*0.45*	*0.85*
GO:0006355	*regulation of transcription, DNA-templated*	*9.92%*	*-2.6576*	*0.41*	*0.9*
GO:0060255	*regulation of macromolecule metabolic process*	*11.72%*	*-7.0004*	*0.51*	*0.67*

GO:0019222	*regulation of metabolic process*	*11.94%*	*-5.8539*	*0.65*	*0.67*
GO:0050794	*regulation of cellular process*	*18.84%*	*-5.0937*	*0.6*	*0.76*
GO:1903506	*regulation of nucleic acid-templated transcription*	*9.97%*	*-2.9469*	*0.41*	*0.89*
GO:0009889	*regulation of biosynthetic process*	*10.83%*	*-4.3382*	*0.53*	*0.86*
GO:0010556	*regulation of macromolecule biosynthetic process*	*10.75%*	*-4.4401*	*0.48*	*0.89*
GO:0051171	*regulation of nitrogen compound metabolic process*	*10.93%*	*-6.4908*	*0.52*	*0.86*
**GO:0042221**	**response to chemical**	**3.07%**	**-2.6162**	**0.81**	**0.46**
GO:0051716	*cellular response to stimulus*	*9.56%*	*-3.384*	*0.74*	*0.63*
GO:0006281	*DNA repair*	*2.23%*	*-1.4724*	*0.59*	*0.9*
GO:0033554	*cellular response to stress*	*2.97%*	*-1.4737*	*0.75*	*0.81*
GO:0006974	*cellular response to DNA damage stimulus*	*2.36%*	*-1.7122*	*0.75*	*0.51*
GO:0080134	*regulation of response to stress*	*0.34%*	*-1.684*	*0.65*	*0.62*
**GO:0044271**	**cellular nitrogen compound biosynthetic process**	**22.50%**	**-1.7328**	**0.71**	**0.47**
**GO:0018205**	**peptidyl-lysine modification**	**0.36%**	**-1.3134**	**0.83**	**0.48**

***mlk1/2/3* ZT14**

GO-term ID	Description	Frequency	Log_{10} p-value	Uniqueness	Dispensability
**GO:0051276**	**chromosome organization**	**1.48%**	**-2.7852**	**0**	**0**
GO:0007064	*mitotic sister chromatid cohesion*	*0.05%*	*-1.7645*	*0*	*0.54*
GO:0006325	*chromatin organization*	*0.67%*	*-1.3072*	*0*	*0.53*

***mlk1/3/4* ZT14**

GO-term ID	Description	Frequency	Log_{10} p-value	Uniqueness	Dispensability
**GO:0009628**	**response to abiotic stimulus**	**0.57%**	**-1.857**	**0.91**	**0**
**GO:0009987**	**cellular process**	**63.78%**	**-2.1512**	**0.97**	**0**
**GO:0010629**	**negative regulation of gene expression**	**0.78%**	**-1.644**	**0.62**	**0**
**GO:0050896**	**response to stimulus**	**12.21%**	**-1.8962**	**0.94**	**0**

**GO:0051276**	**chromosome organization**	**1.48%**	**-2.1175**	**0.86**	**0**
GO:0006325	*chromatin organization*	*0.67%*	*-1.308*	*0.86*	*0.53*
**GO:0065007**	**biological regulation**	**20.50%**	**-2.4425**	**0.94**	**0**
**GO:0009058**	**biosynthetic process**	**31.61%**	**-1.6655**	**0.88**	**0.02**
**GO:0044249**	**cellular biosynthetic process**	**30.05%**	**-2.762**	**0.65**	**0.06**
GO:1901576	*organic substance biosynthetic process*	*30.37%*	*-2.2013*	*0.68*	*0.66*
GO:0018130	*heterocycle biosynthetic process*	*17.39%*	*-1.7144*	*0.68*	*0.53*
GO:0019438	*aromatic compound biosynthetic process*	*16.95%*	*-1.5114*	*0.68*	*0.53*
GO:1901362	*organic cyclic compound biosynthetic process*	*17.87%*	*-1.752*	*0.7*	*0.54*
**GO:1901360**	**organic cyclic compound metabolic process**	**30.32%**	**-1.301**	**0.86**	**0.07**
**GO:0071704**	**organic substance metabolic process**	**58.36%**	**-1.5157**	**0.89**	**0.08**
**GO:0006807**	**nitrogen compound metabolic process**	**38.74%**	**-1.4377**	**0.88**	**0.09**
**GO:0044237**	**cellular metabolic process**	**53.06%**	**-2.2358**	**0.83**	**0.18**
**GO:0046483**	**heterocycle metabolic process**	**29.66%**	**-1.3439**	**0.81**	**0.25**
**GO:0097305**	**response to alcohol**	**0.06%**	**-1.5686**	**0.91**	**0.29**
**GO:0019222**	**regulation of metabolic process**	**11.94%**	**-3.5229**	**0.56**	**0.38**
GO:0050794	*regulation of cellular process*	*18.84%*	*-1.5935*	*0.52*	*0.76*
GO:0050789	*regulation of biological process*	*19.37%*	*-3.0804*	*0.57*	*0.67*
GO:0080090	*regulation of primary metabolic process*	*11.68%*	*-2.6003*	*0.49*	*0.87*
GO:0009889	*regulation of biosynthetic process*	*10.83%*	*-1.7986*	*0.4*	*0.86*
GO:0031323	*regulation of cellular metabolic process*	*11.66%*	*-1.9957*	*0.45*	*0.87*
GO:0031326	*regulation of cellular biosynthetic process*	*10.82%*	*-1.8665*	*0.38*	*0.86*
GO:0060255	*regulation of macromolecule metabolic process*	*11.72%*	*-2.8069*	*0.47*	*0.62*
GO:0051171	*regulation of nitrogen compound metabolic process*	*10.93%*	*-2.7959*	*0.49*	*0.86*
